# Gastrointestinal stromal tumor in perforated Meckel’s diverticulum: a case report and literature review

**DOI:** 10.1186/s40792-020-01038-x

**Published:** 2020-10-07

**Authors:** Naoki Hashizume, Saki Sakamoto, Suguru Fukahori, Shinji Ishii, Nobuyuki Saikusa, Yoshinori Koga, Naruki Higashidate, Shiori Tsuruhisa, Hirotomo Nakahara, Yoshiaki Tanaka, Minoru Yagi

**Affiliations:** 1 Department of Pediatric Surgery, Kurume University School of Medicine, 67 Asahimachi, Kurume, Fukuoka 830-0011 Japan; 2 Division of Medical Safety Management, Kurume University Hospital, 67 Asahimachi, Kurume, 830-0011 Fukuoka Japan

**Keywords:** Gastrointestinal stromal tumor, Meckel’s diverticulum, Perforation

## Abstract

**Introduction:**

Gastrointestinal stromal tumor (GIST) is rare neoplasms of the gastrointestinal tract associated with high rates of malignant transformation. GIST has been found largely in the stomach, small bowel, colon and rectum, and esophagus, but about 5% are found in other locations. We herein report a 56-year-old woman with a GIST in perforated Meckel's diverticulum. After encountering this patient, a review of the literature found reports of 18 similar patients.

**Case presentation:**

A 56-year-old woman diagnosed with galactosialidosis (β-galactosidase-neuraminidase deficiency) presented with vomiting. On contrast-enhanced computed tomography, peritonitis due to perforation of the intestine was diagnosed based on the free air and dilated loop of the small bowel. Laparotomy revealed perforation of Meckel’s diverticulitis located 50 cm from the ileocecal valve. Partial resection of the ileum, including the diverticulum, and end-to-end anastomosis of the small intestine were performed. Regarding the pathological findings, the edge of the diverticulum wall consisted of a solid mass measuring 1.0 cm in size, and the tumor cells were spindle-shaped with 1 mitosis present per 50 high-power fields. The diagnosis was established as GIST of the Meckel's diverticulum. The postoperative period was uneventful. Follow-up at two years revealed no evidence of recurrence.

**Conclusion:**

GIST in perforated Meckel's diverticulum is very rare. The potential for the coexistence of GIST or other tumor should be considered in the treatment of perforated Meckel's diverticulum.

## Introduction

Gastrointestinal stromal tumor (GIST) is rare neoplasms of the gastrointestinal stromal tract associated with high rates of malignant transformation. GIST has been found largely in the stomach, small bowel, colon and rectum, and esophagus, but about 5% are found in other locations [1].

Meckel’s diverticulum is the most common congenital abnormality of the GI tract. The incidence of Meckel diverticulum in the general population is 1% [2]. Common complications presenting in adults include bleeding, obstruction, diverticulitis, and perforation. About 30% of all Meckel’s diverticula contain ectopic or abnormal tissue [2, 3]. Tumors within Meckel’s diverticulum are rare, with a reported incidence of 0.5% to 3.2% [2, 3]. These tumors are commonly benign, like leiomyomas, angiomas, and lipomas, and the majority of malignant neoplasms are adenocarcinoma, sarcoma, and carcinoid tumours, with few being GISTs.

We herein report a case of GIST in perforated Meckel's diverticulum along with the largest collection of previously reported cases of GIST in perforated Meckel's diverticulum, gathered from the literature.

## Case report

**Fig. 1:**
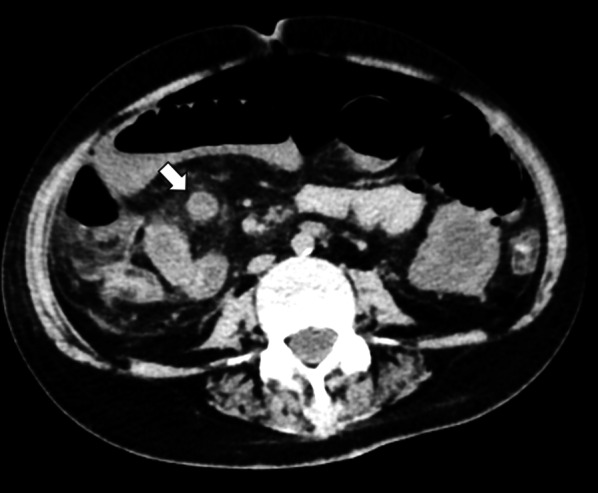
Computed tomography coronal view image obtained free air and a right quadrant heterogeneous mass within the loop of ileum (close arrows)

A 56-year-old woman diagnosed with galactosialidosis (β-galactosidase-neuraminidase deficiency) presented with vomiting. At admission, her general condition was poor, with a pulse rate of 90 bpm, blood pressure 110/60 mmHg, and oxygen saturation 100% without oxygen flow. On a physical examination she showed muscular defense over the whole abdomen. The laboratory test results on admission were as follows: red blood cell count, 488 × 10^6^ cells/μL; hemoglobin 13.5 g/dL; leukocyte count, 6400 cells/μL; platelet count, 15.6 × 10^4^ cells/μL; aspartate aminotransferase, 20U/L; alanine aminotransferase, 7 U/L; and C-reactive protein, 12.1 mg/dL. Abdominal X-ray photograph revealed a large amount of gas over most of the abdomen. Peritonitis due to perforation of the intestine was diagnosed based on the free air and dilated loop of the small bowel found on contrast-enhanced computed tomography (Fig. 1).

**Fig. 2:**
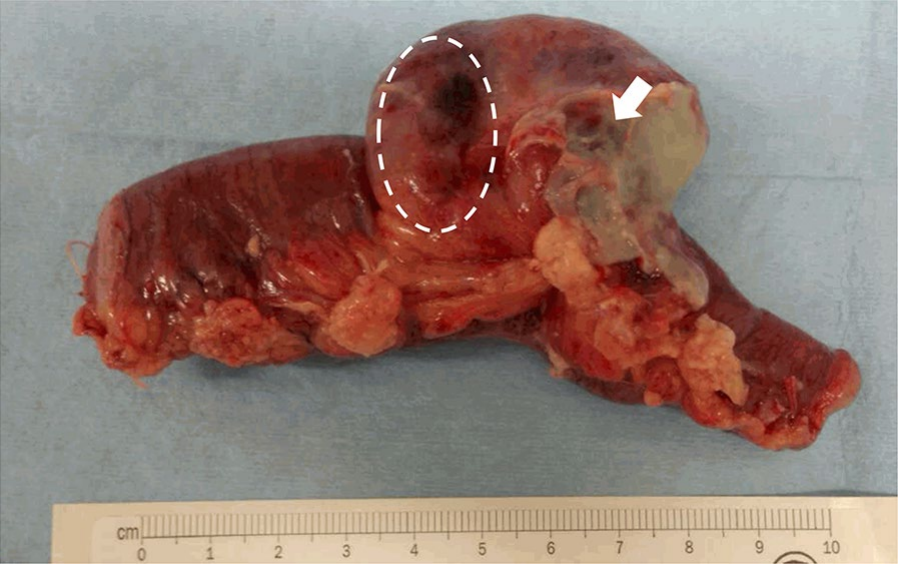
Resected Meckel's diverticular mass with 5 cm margin of small bowel on either side of the lesion. The location of GIST was in the dotted line and perforation position of Meckel's diverticulum was located by the arrow

Laparotomy revealed perforation of the Meckel’s diverticulitis located 50 cm from the ileocecal valve. Partial resection of the ileum, including the diverticulum, and end-to-end anastomosis of the small intestine were performed (Fig. 2).

**Fig. 3:**
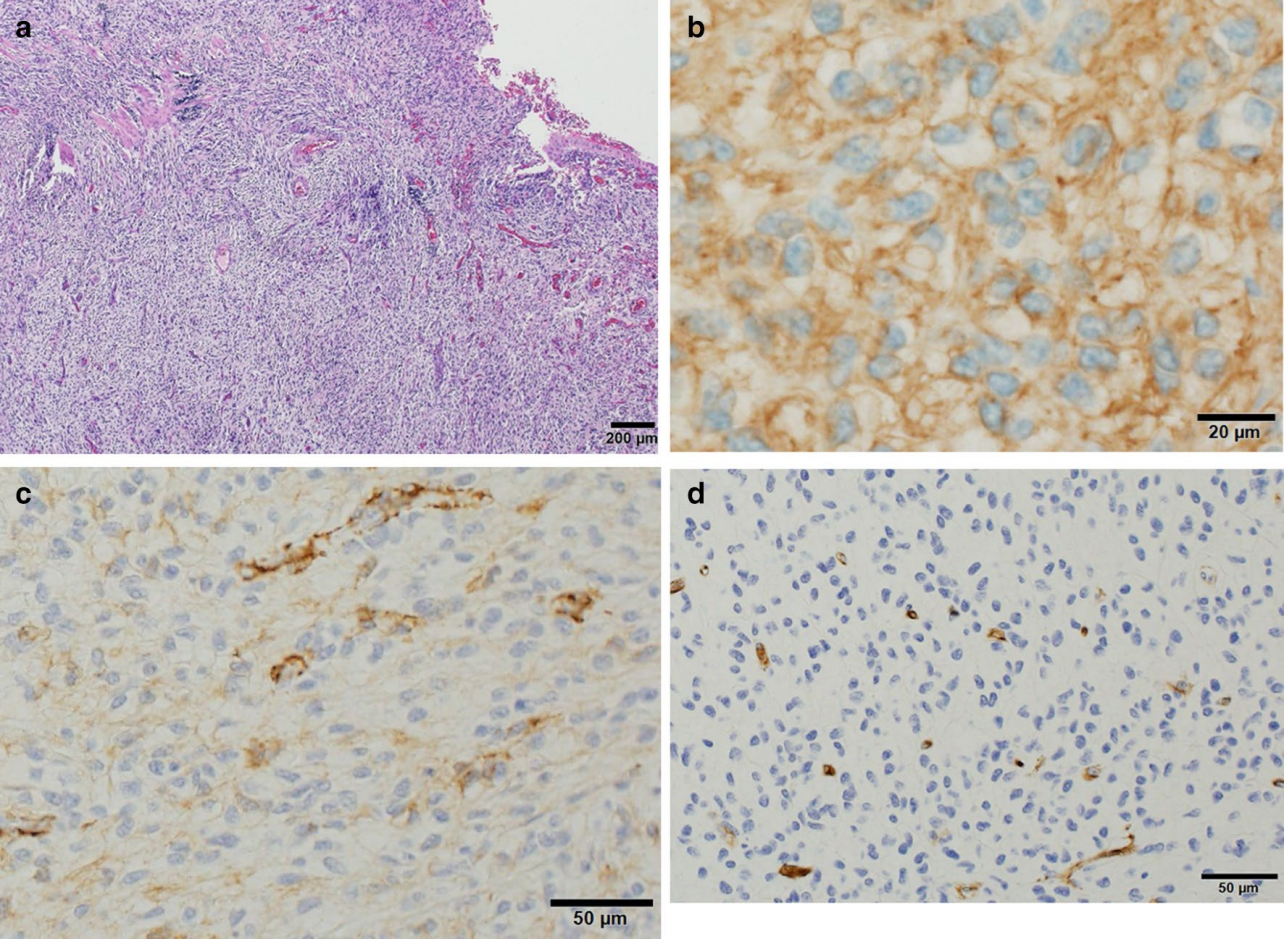
A histopathologic examination revealed the tumor cells were spindle and 1 mitosis was present in 50 high-power fields (**a**). Immunohistochemistry results were as follows: the cytoplasm was stained positive for c-kit (**b**), α-smooth muscle actin (**c**). The cytoplasm was stained negative for CD34 (**d**)

Regarding the pathological findings, ectopic gastric mucosa was not observed in the diverticulum. The edge of the diverticulum wall consisted of a solid mass measuring 1.0 cm in size, and the tumor cells were spindle-shaped with 1 mitosis present per 50 high-power fields (Fig. 3a). The perforation site of the diverticulum was separated from the tumor nodule. The immunohistochemistry findings were as follows: the cytoplasm was stained positive for c-tyrosine kinase receptor (kit) (Fig. 3b) and α-smooth muscle actin (Fig. 3c) and stained negative for CD34 (Fig. 3d). The Ki–67 index was 1.6%. The diagnosis was established as GIST of Meckel's diverticulum.

As the patient had been diagnosed with galactosialidosis and was intolerant to imatinib and because the perforation of the diverticulum was not associated with the tumor nodule, imatinib was not administered. The postoperative period was uneventful. Follow-up at two years revealed no evidence of recurrence.

## Discussion

For many patients, GIST may be detected as an incidental finding during an evaluation of nonspecific symptoms. GIST most commonly occurs in the stomach or small bowel [4]. Symptoms tend to arise only when tumors reach a large size or are in a critical anatomic location; indeed, most symptomatic patients present with tumors larger than 5 cm in their largest dimension. Symptoms at presentation may include abdominal pain, abdominal mass, nausea, vomiting, anorexia, and weight loss. About 10% to 30% of GISTs progress to malignancy. GISTs occurring outside of the stomach are associated with a higher malignant potential [1, 5]. Metastatic spread to lymph nodes and other regions via lymphatics is very rare.

Gastrointestinal stromal tumors are mesenchymal tumors that originate from the interstitial cells of Cajal. The majority of GISTs are positive for the tyrosine kinase receptor Kit, although Kit positivity is no longer required for the diagnosis [6]. The most reliable prognostic factors for risk classification are the size of the primary tumor and the mitotic index, which measures the proliferative activity of the cells [3]. The approaches to treating GISTs are resection of primary low-risk tumors and resection plus imatinib for high-risk primary or metastatic tumors. According to Johnsue’s classification [7], tumor rupture merits assignment to the high-risk category, and any patient presenting with a perforated GIST should receive imatinib, regardless of the mitotic count, as there is a possibility of tumor cells being disseminated into the peritoneal cavity after perforation. It was supposed that the cause of perforation was determined by a superficial necrosis of the mucosa and relative ulceration for the development of GIST at the sub mucosal which determines compression of the surface layer.

Meckel’s diverticulum is a true diverticulum containing all layers of the intestinal wall and most commonly arises from the antimesenteric aspect of the ileum, proximal to the ileocecal valve. Tumors within Meckel’s diverticulum are rare. Thirunavukarasu et al. reported that an underlying reason for the difference in tumor distribution between the small bowel and Meckel's diverticulum has not been described. However, tumors in a Meckel’s diverticulum are approximately 70 times more common than ileal cancers on a ‘per length of bowel at risk’ basis when the prevalence and length of Meckel's diverticulum are taken into account. Given the increasing risk with age and high possibility of curative resection with negligible operative mortality, incidental Meckel's diverticulum is best treated with resection [8].

A literature search was performed using the electronic database “PubMed” for all patient reports in the English literature with GIST in Meckel's diverticulum using the search terms “gastrointestinal stromal tumor”, “perforated” and “Meckel's diverticulum”. Relevant data were extracted from all primary reported patients. Patients included in multiple reports were used only once for the analysis. All patient data were combined to create this report. There have been 18 cases of GIST in perforated Meckel's diverticulum, as shown in Table 1 [9–24]. No recurrences were reported. The clinical features of the current case were consistent with those previously reported, including the gender, age, operative findings, treatment, follow-up, tumor size, mitotic figures and risk classification [7]. These patients were 11 men and 6 women, with gender not mentioned in 1 case. The mean age at presentation was 60 years old, ranging from 23 to 86 years old. The tumors ranged in longitudinal length from 1 to 8 cm. In the review, almost all of the patients required small bowel resection. Since 2013, seven patients underwent imatinib chemotherapy after their operation [19–25]. The tumors ranged in longitudinal length from 1 to 14 cm. The tumor sizes in 4 patients were under 2 cm, those in 12 patients were over 2 cm, and those in 4 patients were unknown. Low or < 5 mitoses were present in 50 high-power fields in 8 patients, and high or ≥ 5 mitoses were present in 50 high-power fields in 6 patients; the findings in 3 patients were unknown. The mitotic figure National Institutes of Health risk classifications were as follows: two patients had a very low risk, six had a low risk, four had an intermediate risk, two had a high risk, and four had an unknown risk [6]. One patient died two months after surgery.

**Table 1:** Reported cases of gastrointestinal stromal tumor in perforated Meckel's diverticulum (*n* = 18)

Report	Year	Gender	Age	Operative findings	Treatment	Follow up	Tumor size (cm)	Mitotic figure (/50HPFs)	Risk classification*
Fruhauf [9]	2002	M	61	Peritonitis	NR	NR	NR	High	–
Szentpáli [10]	2004	M	70	Peritonitis	SBR	Alive 3Y	1.5	Low	Very low
Hager [11]	2005	M	75	Peritonitis	NR	NR	NR	NR	–
Woolf [12]	2009	M	59	Peritonitis, haemorrhage	SBR	Alive	4.5	< 5	Low
Caricato [13]	2010	M	65	Peritonitis	SBR	Alive 2Y	4.5 × 3.7 × 3.5	1	Low
Chou [14]	2011	F	76	Peritonitis	SBR	NR	3.2	6	Intermitted
Dogrul [15]	2011	F	86	Peritonitis	SBR	Dead 2 months	8	High	High
Sozen [16]	2012	F	62	Peritonitis	SBR	NR	2.5	6	Intermitted
Mitula [17]	2012	F	63	Peritonitis	SBR	Alive 6 months	14	High	High
Goyal [18]	2013	M	23	Abscess	SBR, imatinib	Alive 1Y	4.8 × 4.2 × 4.2	< 5	Low
Fernandez [19]	2013	NR	NR	Peritonitis	SBR, imatinib	NR	NR	NR	–
Nayak [20]	2015	M	50	Peritonitis	SBR	NR	NR	NR	–
Kiliç [21]	2015	F	37	Peritonitis	SBR, imatinib	NR	4.5 × 2.5 × 2.5	10	Intermitted
Ikemura [22]	2015	M	82	Peritonitis	SBR, imatinib	Alive 22 months	2.5	1	Low
Hosamani [23]	2016	M	40	Peritonitis	SBR, imatinib	Alive 5Y	3 × 1 × 1	2	Low
Omerza [24]	2016	M	55	Peritonitis	SBR, imatinib	NR	3.5 × 8	< 5	Intermitted
Miyata [25]	2016	M	62	Peritonitis	SBR, imatinib	NR	4	0	Low
Our case	2018	F	56	Peritonitis	SBR	Alive 1Y	1 × 1	< 5	Very low

The risk classification guideline of the National Institutes of Health

*M* male, *F* female, *NR* not reported, *SBR* small bowel resection

The exact mechanism of the perforation was explained tumor necrosis, making ulcer for the pressure of tumor, bowel obstruction and tumor invasion into the muscularis propria, replacing the gut wall [22]. Although the mechanism of perforation in this case were not clear, it was suspected that increased intraluminal pressure and inflammation secondary to distal bowel obstruction due to the tumor occurred the perforation of the Meckel’s diverticulum.

## Conclusion

In conclusion, GIST in perforated Meckel’s diverticulum is very rare. The potential for the coexistence of GIST or other tumor should be considered in the treatment of perforated Meckel's diverticulum.

## Data Availability

All data generated during this study are included in this published article.

## Abbreviations

GIST: Gastrointestinal stromal tumor
kit: Tyrosine kinase receptor
